# Choice alters *Drosophila* oviposition site preference on menthol

**DOI:** 10.1242/bio.20136973

**Published:** 2013-09-29

**Authors:** Dehbia Abed-Vieillard, Jérôme Cortot, Claude Everaerts, Jean-François Ferveur

**Affiliations:** Centre des Sciences du Goût et de l'Alimentation, UMR 6265 CNRS, UMR 1324 INRA, Université de Bourgogne, Dijon, 6, Bd Gabriel, F-21000 Dijon, France

**Keywords:** Egg-laying behaviour, Aversive behaviour, Menthol, Caffeine, Sucrose

## Abstract

Food choice and preference relies on multiple sensory systems that are under the control of genes and sensory experience. Exposure to specific nutrients and nutrient-related molecules can change food preference in vertebrates and invertebrates. For example, larval exposure of several holometabolous insects to menthol can change their adult response to this molecule. However, studies involving *Drosophila melanogaster* exposure to menthol produced controversial results due maybe to methodological differences. Here, we compared the oviposition-site preference of wild-type *D. melanogaster* lines freely or forcibly exposed to menthol-rich food. After 12 generations, oviposition-site preference diverged between the two lines. Counterintuitively, menthol ‘forced’ lines showed a persistent aversion to menthol whereas ‘free choice’ lines exhibited a decreased aversion to menthol-rich food. This effect was specific to menthol since the ‘free choice’ lines showed unaltered responses to caffeine and sucrose. This suggests that the genetic factors underlying *Drosophila* oviposition site preference are more rapidly influenced when flies have a choice between alternative sources compared to flies permanently exposed to the same aversive substance.

## Introduction

Food preference depends on the interaction between environmental and physiological cues informing the animal on its degree of satiety (Melcher et al., 2007; Kent and Worsley, 2009). This information is processed by sensory and physiological systems depending on complex networks of genes (Greenspan, 2001; Brown and van der Ouderaa, 2007). During life, food preference can be affected both by sensory experience and metabolism in vertebrates (Yeomans et al., 2008; Stewart et al., 2011) and in insects (Ogueta et al., 2010; Fougeron et al., 2011). Food preference in young vertebrates can be influenced by factors such as the mother's diet during embryonic and foetal development (Mennella et al., 2001; Bertin et al., 2012). The transmission of food preferences in insects may also depend on early sensory learning, thus leaving an ‘imprinted’ trace into adult life (Papaj and Prokopy, 1989; Zhang et al., 2005; Dukas, 2008; Gerber et al., 2009). In holometabolous insects (i.e. exhibiting a ‘complete metamorphosis’; for example, Hymenoptera and Diptera), larval exposure to menthol can reduce aversion to this substance in resulting adults compared to naive insects (Thorpe, 1939; Alloway, 1972). However, this hypothesis remains uncertain in *Drosophila melanogaster* (Barron and Corbet, 1999; Barron and Corbet, 2000).

Animals raised in the laboratory are exposed to a constant diet contrarily to wild-type animals that have a broader choice of food sources in nature. Laboratory strains of *D. melanogaster* exposed to alternative food display no switch in food preference but increased orientation to novel food cues (Barron and Corbet, 2000). However, the effect of repeated exposure, generation after generation, to constant or alternative food sources remains unknown. Here, we investigated the oviposition-site decision response to menthol of wild-type *D. melanogaster* lines forcibly kept either on menthol-rich food or on plain food only (‘forced lines’) or presented to a choice between the two types of food. In the last case, we took advantage of the short generation duration of this species to establish ‘choice lines’, showing either a preference, or an aversion, to oviposit on menthol. Many *Drosophila* studies have used experimental selection of complex behaviour to identify the gene(s) underlying behavioural response. For example, microarrays performed on choice lines have pinpointed some of the genes underlying geotactism (Toma et al., 2002), aggressive behaviour (Dierick and Greenspan, 2006) or sex pheromone discrimination (Houot et al., 2012). Genetic basis for the choice of site and the decision to oviposit were investigated in *D. melanogaster* for many years (Takamura and Fuyama, 1980; Allemand and Boulétreau-Merle, 1989; Yang et al., 2008; Miller et al., 2011). In our laboratory, we have maintained more than 50 generations of wild-type *D. melanogaster* lines consistently on either menthol-rich food (‘forced lines’), or a diet based on their initial oviposition preference phenotype (attraction/aversion, ‘choice lines’). Our data suggest that oviposition-site decision response is differently affected between forced and choice lines.

## Materials and Methods

### Flies

Two wild-type strains of *Drosophila melanogaster* Meigen were used: the widely used laboratory strain Canton-S (CS) and the Dijon2000 strain (Di2) established in 2000. Since initial data obtained with the two strains showed no significant differences in behavioural response to menthol, they were pooled. Subsequent experiments were performed with the Di2 strain. Flies were raised on a yeast–cornmeal–agar medium and kept at 24±0.5°C with 65±5% humidity on a 12-hr light:12-hr dark cycle. All experiments were performed under similar conditions. Flies were collected and sexed 0–4 hour after emergence under light CO_2_ anaesthesia and held until 4 to 5 day-old in groups of 30 same-sex individuals in glass vials with fresh plain food. Unless mentioned, all other flies including those of parental lines were kept in a menthol-free environment.

### Menthol and food preparation

We used pure racemic menthol (M0321, TCI, Japan). A 250 mg/ml solution was prepared by dissolving menthol in 90% (*v/v*) ethanol and kept at 4°C. Menthol solution was added to fresh lab food and is designated hereafter as menthol-food (M-food). A similar volume of ethanol (90% *v/v*) was added to the control diet menthol-free food (Plain-food  =  P-food). Egg-laying food devices (see hereinafter) were used immediately or kept at 4°C to be used until two days later. To assess the optimal proportion of menthol to be used in our tests, we performed preliminary multiple-choice tests using a range of menthol containing food, and measured the number of eggs laid on each type of food. Concentrations ranging from 0 to 0.01% induced no food preference (MPI = 0; see hereinafter for MPI formula), whereas an aversive effect was detected from 0.1% menthol (MPI = −0.4, corresponding to 70% eggs laid on P-food and 30% on M-food). At the menthol concentration of 0.5%, females rarely oviposited on M-food (MPI = −1). All the experiments presented here were thus carried out with a menthol concentration of 0.1%. To assess the specificity of the choice procedure (carried out with menthol), we also measured egg-laying behaviour of F24 and F25 choice-line females on food enriched either with caffeine (16 mM) (Sigma–Aldrich, USA) or sucrose (10 mM) (Sigma–Aldrich, USA). To obtain and test enough individuals, we carried this experiment a few generations after our choice procedure had resumed with F24+F25 flies (supplementary material Fig. S1).

### Oviposition behaviour

**Fig. 1.**
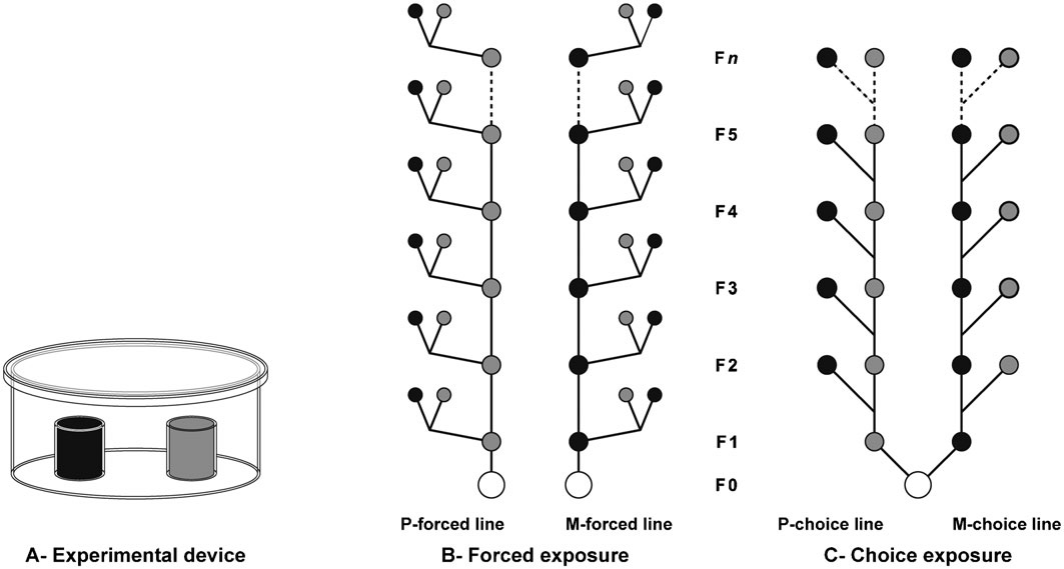
Procedures of menthol exposure. Schematic representation of the (A) experimental device used for egg-laying behaviour and (B,C) the two procedures used to expose *Drosophila melanogaster* wild-type lines to food enriched with 0.1% menthol (M-food). (A) Females laid eggs in a device made with a petri dish covered with a glass dish and containing two egg-laying sites either filled with M-food or with P-food (see Materials and Methods). (B) Lines (issued from parental lines; empty circles) were either continuously raised on plain food (P-food; P-forced line, shaded circles) or on M-food (M-forced line, filled circles). At each generation (from F1 to F*n*), flies of the P- and M-forced food were tested for choice between M- and P-food (pairs of circles connected on either side). (C) Lines were established on their preference when tested in dual-choice between P- and M-food. On the left side, eggs laid on P-food were repeatedly used to generate the P-choice line until the F*n* generation. In parallel (right side), eggs laid on M-food were used to generate the M-choice line until the *Fn* generation. Oviposition site preference was measured in the two procedures.

Groups of 4–5-day-old flies composed of 25 adult females and 25 adult males (previously CO_2_-anaesthetized) were kept overnight in a dual-choice egg-laying device (Fig. 1A). This device consisted of a petri dish (10 cm diameter) covered with a glass dish (300 ml) and containing two egg-laying sites either filled with M-food or with P-food. Each site was made of a plastic cylinder (2 cm diameter, 2 cm height) closed at its bottom by a cap and filled with 5 g of food. Both sites were fixed with a drop of glue (Patafix UHU, Germany) at a distance of 2.5 cm from each other. This arrangement insured that no oviposition behaviour difference was observed in control experiments with the two sites filled with P-food (Wilcoxon test, *p* = 0.563, *n* = 20).

The numbers of (*i*) fertilized eggs laid and (*ii*) surviving females were counted after 12 hours. This allowed us to determine the average number of eggs laid per female (egg per female  =  EPF). The comparison of the numbers of eggs laid on M-food (Nm) *vs* P-food (Np) was used to compute the menthol preference index (MPI = [Nm−Np]/[Nm+Np]). In theory, MPI can vary between −1 (strong aversion against menthol) and +1 (strong attraction to menthol).

To assess age-dependent effects, the egg-laying behaviour of F12 females (after 12 generations of choice selection procedure) was measured daily during their first 5 days of adult life using the parameters described above. Since the number of eggs laid on days 4 and 5 were relatively low, the data obtained for the two days were pooled to permit statistical analysis.

To test a possible effect on female fecundity, we recorded, between F0 and F12, the total number of eggs laid by control, P-forced and M-forced females on P-food or on M-food during 12 h (25 females × 20 replicates).

### Exposure procedures

Two distinct experimental procedures were designed to assess the effect of forced *vs* choice exposure to menthol. The **‘forced’ procedure** consisted to raise individuals during their complete development, generation after generation, either on M-food (M-forced line; filled circles on Fig. 1B) or P-food (P-forced line; shaded circles). The egg-laying behaviour of adult females resulting from these two lines was measured in the ‘M- *vs* P-food’ dual-choice, at several generations until F52 (supplementary material Figs S1, S2).

The **‘choice’ procedure** consisted in keeping the progeny left either on M- or on P-food, generation after generation on that food. More precisely, parental flies (F0; empty circles) had the choice to lay eggs in the M- *vs* P-food dual-choice device. Only the eggs laid on M-food were used to initiate the F1 ‘choice M-line’ (right arm of Fig. 1C). Once adults, these F1 flies had the choice to lay eggs and only those laid on M-food were kept to initiate the next choice M-line generation. To avoid a statistical bias related to small samples, we only kept eggs on food devices when the number of eggs laid on M-food represented at least ¼ of the total number of eggs (Nm≥25%[Nm+Np]). In parallel, a ‘choice P-line’, initiated with eggs laid on P-food, was established using a similar procedure (with Np≥[75%×(Nm+Np)]; left arm of Fig. 1C). Oviposition behaviour was measured during a period encompassing 58 generations, but could not be carried out at all generations. For the sake of clarity, we show the data obtained for the F0–F12 generations (with a continuous experimental selection) in the main part of our study (except for the specificity experiment performed with F24+F25 females) whereas the data corresponding to further generations (F19–F58) are shown in supplementary material Figs S1 and S2. For technical reasons, the choice procedure was interrupted during several periods corresponding to the F13–F19 generations (oviposition behaviour was nevertheless checked in F17 choice lines) and to the F26–F36 generations. Therefore, the choice procedure was carried out for F1–F12, F20–F25 and F37–F48 generations. During the interruption of the choice procedure, P- and M-choice lines were, respectively, kept on P- and M-food at 18°C. Oviposition behaviour was finally checked in F58 choice-line females.

To control the effect of each experimental procedure, parental strains (unexposed ‘control lines’), kept in similar laboratory conditions, were tested at the beginning and during the course of our experiments. Data from all lines (forced or choice on M-, P-food and controls) were obtained by testing at least 4 sublines per line (each subline was maintained in a separate food vial) and each generation was initiated by at least 2 replicates per subline. To avoid potential bottlenecks in choice lines, a generation was established with at least 100 eggs per vial.

### Statistics

All statistical analyses were performed with XLSTAT 2012 software (Addinsoft, XLSTAT 2012, Data analysis and statistics with Microsoft Excel, Paris, France). In choice experiments, the number of eggs found on oviposition sites was compared using Wilcoxon signed ranked test. Intra-generational difference in the MPIs (EPFs) of P- and M-females were assessed using Mann–Whitney test, whereas MPIs (and/or EPFs) throughout F0–F12 generations were compared using Kruskal–Wallis test (*p*-value based on a Monte Carlo computation) completed by Conover–Iman multiple pairwise comparisons (two-tailed with Bonferroni correction, level *p* = 0.05). In addition, to visualize the change of menthol preference from F0 to F12 in each experimental procedure, we used the EPFs on M- and on P-food in the two lines to build a contingency table (19×2 for forced exposure and 25×2 for choice exposure) used to perform a Chi-square analysis with computation of the adjusted residuals. Adjusted residual test provides a fair indication of the importance of the cells to the ultimate Chi-square value and reveals how far the observed values are from the expected ones. They have a standard normal distribution (i.e. mean = 0 and SD = 1), and an adjusted residual higher than 1.96 or lower than −1.96 indicates that the observed value is significantly larger, or smaller, than the expected one (Agresti, 2002). For each experimental procedure, adjusted residual values provided an estimation of the ‘Menthol Preference’ that was used as dependant variable together with ‘Generation’ as explanatory variable in a linear regression analysis.

Furthermore, to test the overall effect – with all generations taken into consideration – of each experimental procedure, the MPI and EPF of P- and M-lines were compared with an ANCOVA. MPI data were Arcsin-transformed (MPI+1). MPI and EPF were considered as dependent variables, whereas ‘Food type’ (control, P-, M-food) was considered as a qualitative independent variable and ‘Generation’ as an independent quantitative variable. When the ANCOVA result was significant, we used the Ryan–Einot–Gabriel–Welsch (REGWQ) test to compare food type effect. For choice lines, ANCOVA only included generations with choice procedure: the first analysis was carried out on [F0–F12], the second analysis on ([F0–F12]+[F20–F25]), and the third one on ([F0–F12]+[F37–F46]).

MPI (and/or EPF) during the five first days of adult life of the F12 females, preference index of F24+F25 P- and M-choice females toward caffeine and sucrose and female fecundity (total number of eggs) were assessed using Kruskal–Wallis test as previously described.

## Results

### Oviposition on menthol food

**Fig. 2.**
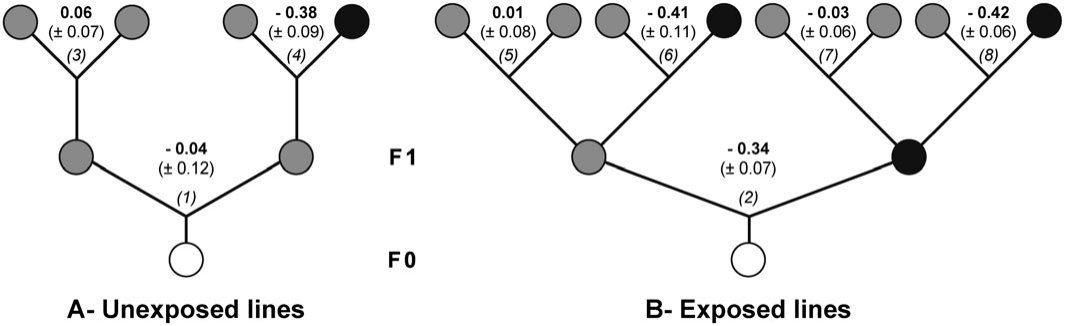
Oviposition site preference on menthol in two differently exposed lines. To test the effect of our experimental design on oviposition-site preference, we compared the preference to P- and M-food (shaded and filled circles, respectively) between two F1 progenies originated from parents (F0; empty circles), which were either (B; exposed line) or not exposed to M-food (A; unexposed line). The menthol preference indices (MPIs; values shown between pairs of circles) were measured between two sites containing either only P-food (no. 1) or P- and M-foods (no. 2). The MPIs of the four F1 sublines (two from unexposed parents  =  nos. 3–4; two from differently exposed parents  =  nos. 5–8) were also measured (*n* = 8–10). The number of eggs laid by females of each line is shown in Table 1.

In all our tests, females had the choice to oviposit either on menthol-rich food (M-food, at 0.1% concentration; see Materials and Methods) or on plain food (P-food). To assess the robustness of our dual-choice test procedure, we compared the menthol-preference indices (MPIs) in flies exposed, or not, to menthol during their complete development (F0; nos. 2 and 1 in Fig. 2). Regardless of prior menthol exposure (in exposed *vs* unexposed lines), MPIs were always negative (−0.38 to −0.42) in ‘M- *vs* P-food’ dual-choice tests (F1; nos. 4, 6, 8) and ‘indifferent’ (−0.03 to +0.06) in ‘P- *vs* P-food’ dual-choice tests (F1; nos. 3, 5, 7). Note that the number of eggs laid per female (EPF) increased by about 50% from F0 (21.5 EPF for nos. 1 and 2; Table 1) to F1 generation lines (30.8 average EPF for nos. 3–8; K_7df_ = 35.31; *p*<0.0001).

**Table 1.**
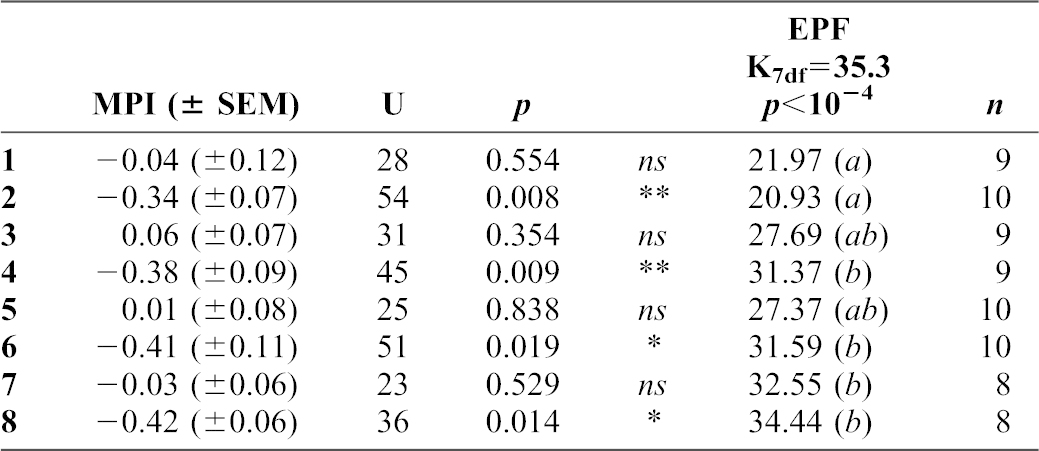
Menthol preference index (MPI) and average number of eggs laid per female (EPF) in F0 and F1 lines. The statistical significance of MPI was assessed with a Wilcoxon signed rank test (*ns*  =  non-significant, **p<0.01, *p<0.05) and the EPFs were compared using a Kruskal–Wallis test (italic letters indicate the results of the multiple pairwise comparison), respectively. The bold numbers of the left column correspond to the lines represented in Fig. 2.

### Forced exposure to menthol

**Fig. 3.**
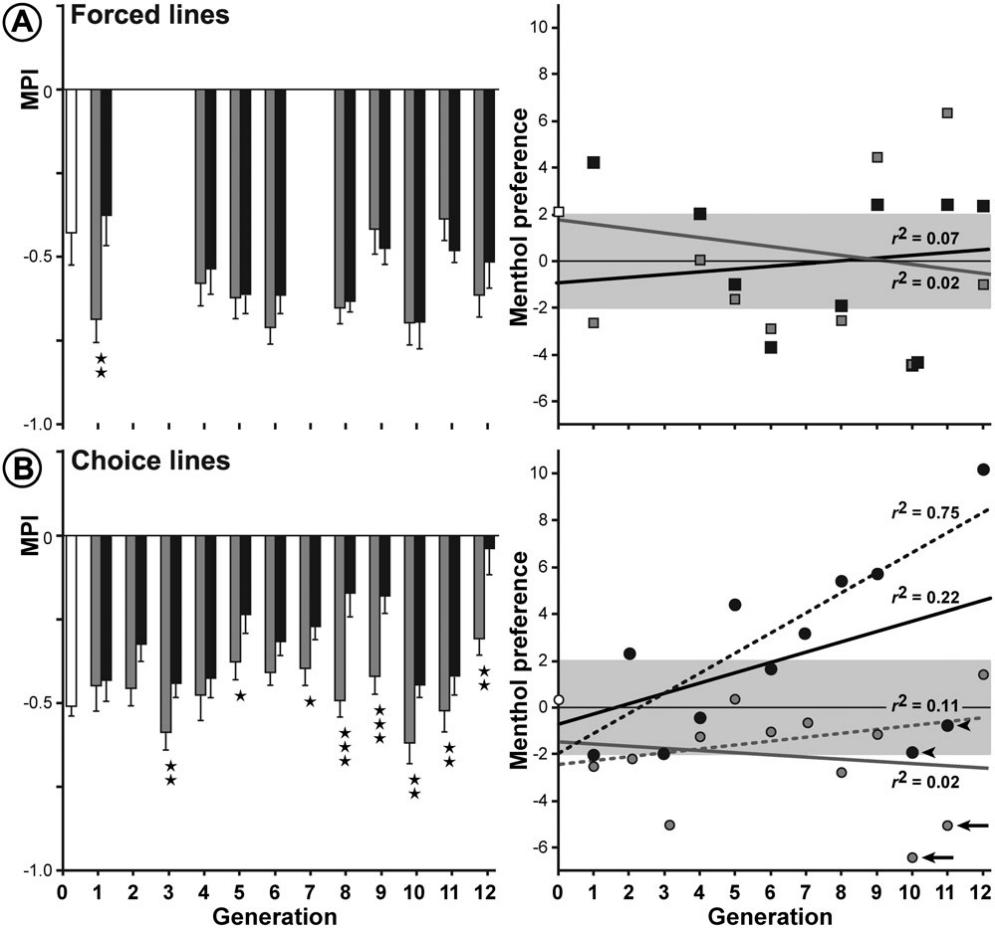
Oviposition site preference on menthol in forced lines (A) and choice lines (B). Left panels: bars represent the mean (± SEM) for the oviposition site preference (MPI) in lines either maintained on P-food (P-line; shaded bars), or on M-food (M-line, filled bars); the control line is shown as empty bars. The performance of both forced lines was measured at several generations between F1 and F12 together with that of the control (unselected) line at the beginning (F0; empty bar). The significance of Mann–Whitney U-tests are indicated as *p<0.05; **p<0.01; ***p<0.001; *n* = 18–38. Right panels: linear regressions of Menthol Preference by Generation in control, P- and M-lines (respectively, empty, shaded and filled symbols). Menthol Preference is significant when higher than 1.96 or lower than −1.96 (outside of the shaded pattern). Regression lines of P- and M-lines (respectively, grey and black) are represented with their corresponding r^2^-values. For choice procedure, arrows and arrowheads pinpoint F10 and F11 flies that negatively impacted the regression similarly in P- and M-choice females. For information, dotted lines (with the corresponding r^2^-values) represent the regression lines when excluding F10 and F11 data.

To determine the long-term effect of the ‘forced’ exposure to menthol, a wild-type strain was permanently kept (during 52 generations) either on M-food (‘M-forced line’) or on P-food (‘P- forced line’; Fig. 1B). In the principal set of data, we focused our study on F1–F12 generations and we measured the ability of females to choose between M- and P-food (MPI; Fig. 3A; supplementary material Fig. S1A) and the number of eggs per female (EPF; supplementary material Fig. S2A) in the two lines.

Except for F1 (Mann–Whitney test U_(29,29)_ = 250; *p* = 0.008), the MPI of the M-forced line was always as negative as the MPI of the P-forced line (*p*-value ranging from 0.365 to 0.841; Fig. 3A, left panel), indicating that flies kept on menthol for many generations maintained their strong initial aversion against this substance. Moreover, no difference was detected between the M- and P-forced lines across the F1 to F12 generations (ANCOVA F_3,443_ = 2.238, *p* = 0.083; supplementary material Table S1) and menthol preference did not change between F1 and F12 generations for P- and M-lines (Fig. 3A, right panel; r^2^ = 0.02 (slope = −0.19) and 0.07 (slope = 0.12); *p* = 0.691 and 0.463, respectively). EPF increased in forced lines during F10–F12 generation; however, without any difference between the M- and P-lines (supplementary material Fig. S2). The fecundity slightly increased after eight generations (F9) in the two forced lines (supplementary material Fig. S3).

In summary, the permanent exposure of lines to menthol did not change the aversive effect induced by this substance and had no major effect on fecundity.

### Choice exposure to menthol

Next, we measured the behavioural effect of menthol exposure on P- and M-choice lines (Fig. 1C, Fig. 3B; supplementary material Fig. S1B). The parental strain used to initiate the choice lines showed a similar aversive MPI as the parental strain used to initiate the ‘forced’ lines. The ‘choice procedure’ differently affected the MPI of the M- and P-choice lines (Fig. 3B, left panel; ANCOVA F_3,637_ = 14.07, *p*<0.0001; supplementary material Table S1). After two generations (F3), the response of M-choice line was generally less negative than in the P-choice line (Mann–Whitney test *p*-value ranging from 0.001 to 0.05, except for F4 – *p* = 0.301 – and F6 – *p* = 0.220). In other words, the strong aversive effect induced by menthol during the first generations gradually disappeared. The progressive change in menthol preference of M-choice line (represented by a relatively low positive correlation: r^2^ = 0.22; slope = 0.45; *p* = 0.101; Fig. 3B, right panel) contrasts with the absence of change in the P-choice line (r^2^ = 0.02; slope = −0.18; *p* = 0.381). Note that the correlation for the M-choice line was negatively impacted by the F10 and F11 generations data (see arrowheads in Fig. 3B, right panel) and P-choice females were similarly impacted at the same two generations (arrows). When the choice procedure resumed (F20–F25 and F37–F46) after some interruption, ‘choice lines’ females showed a non-linear effect with a very strong difference between M- and P-choice at some generations (K_36df_ = 50.998; pairwise comparisons: F20 *vs* F21 and F43 *vs* F45 *p*<0.0001) More rarely, a strong attraction to menthol was noted (at F24 and F25: MPI = +0.5 corresponds to 75% eggs laid on M-food), but these ‘reversed preference’ data points might be outliers, not representative of the general trend. Overall, the three lines differed for their EPF (M-line > P-line > control line: *p*<0.0001; supplementary material Table S1; Fig. S2). The fecundity rapidly increased (between F0 and F1) in the two choice lines and remained elevated at least until F12 (supplementary material Fig. S3).

**Fig. 4.**
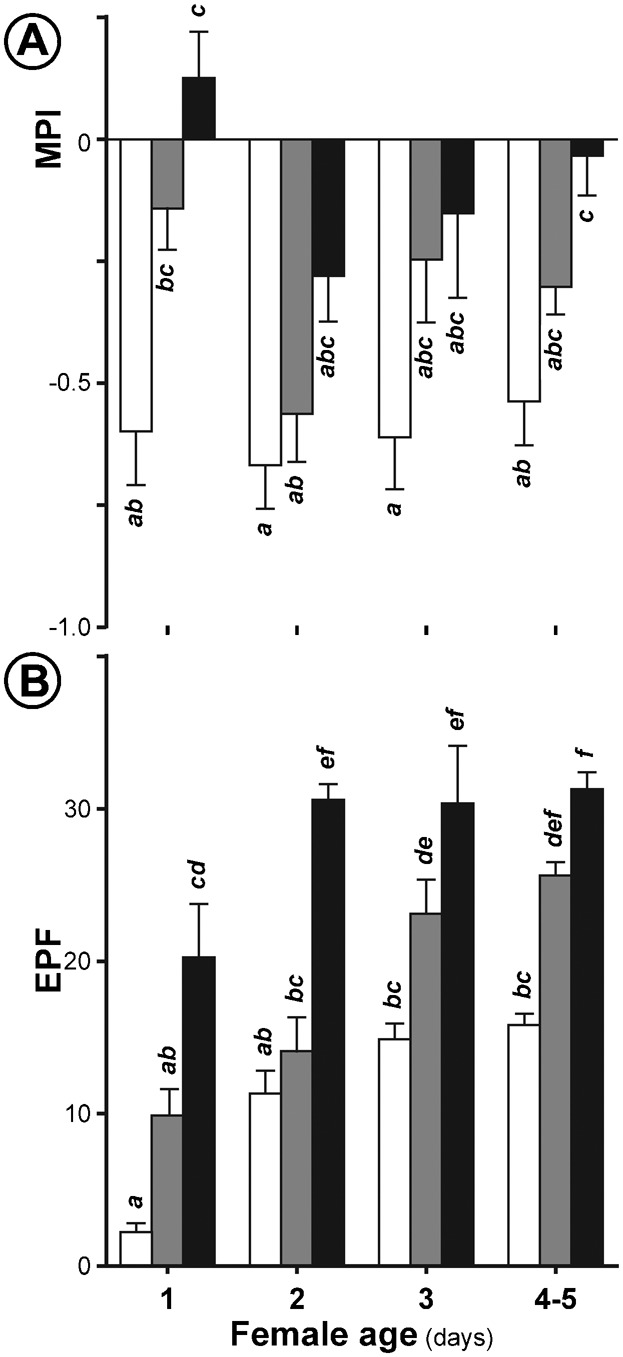
Age-effect on oviposition site preference on menthol in choice lines. Bars represent the mean (± SEM) for the oviposition site preference (MPI; A) and egg number per female (EPF; B) in control, P-choice and M-choice lines (empty, shaded and filled bars, respectively) in the same F12 females of different ages (1–5 days old). Note that 4- and 5-day-old female data were pooled. Letters indicate the significant differences (*p* = 0.05) with a Kruskal–Wallis test (*n* = 8–10).

To assess the stability of the oviposition-choice phenotype, we precisely monitored the MPI and EPF of F12 females during their five first days of adult life. A significant age-related variation of MPI was detected (K_2df_ = 53.404, *p*<0.0001; Fig. 4A). While MPI stayed relatively low from day 1 to day 4–5 for control females, it decreased strongly at day 2 for M-and P-choice females before increasing at 3 days and still more at 4–5 days for M-choice females. EPF also varied with age: more precisely, at days 1 and 2, M-choice line females laid more eggs than same-age P-choice and control females whereas at days 3–5, M- and P-choice females laid more eggs than control females of similar age (K_2df_ = 114.481, *p*<0.0001; Fig. 4B).

### Specificity of the choice exposure procedure

**Fig. 5.**
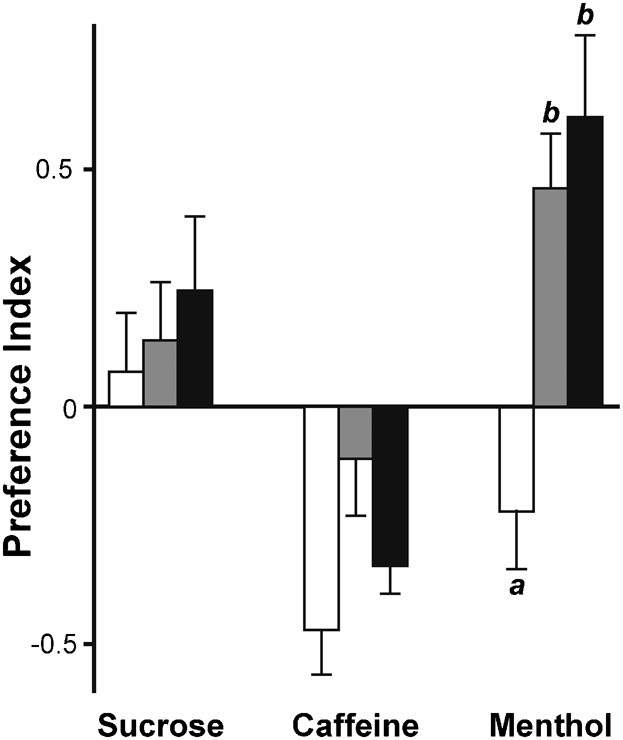
Oviposition site preference to diverse food molecules in choice lines. Bars represent the mean (± SEM) for the oviposition site preference (MPI) in control, P-choice and M-choice lines (empty, shaded and filled bars, respectively) to sucrose, caffeine or menthol. Tests correspond to the F24 and F25 generations. Kruskal–Wallis tests were only significant for menthol (K_2df_ = 18.538, *p*<0.0001) but not for caffeine (K_2df_ = 1.688, *p* = 0.442) or for sucrose (K_2df_ = 0.801, *p* = 0.667). Letters represent the significant differences (*p* = 0.05) with a Kruskal–Wallis test (*n* = 10–23).

To determine the specificity of the choice procedure (performed with menthol-rich food), we measured the response of P- and M-choice lines to caffeine and sucrose. This was carried out with F24+F25 females pooled, four generations after our choice procedure had resumed (supplementary material Fig. S1B, Fig. S2B). Each substance, mixed with food, was presented alongside P-food (Fig. 5). While both P- and M-choice lines showed an altered response to menthol compared to control flies (K_2df_ = 18.538, *p*<0.0001), the response of P- and M-choice lines to caffeine and sucrose was not altered (respectively: K_2df_ = 1.688, *p* = 0.442 and K_2df_ = 0.801, *p* = 0.667).

## Discussion

### Does the ‘choice’ and ‘forced’ procedures reflect the difference between nature and laboratory conditions?

Our results suggest that the behavioural response to menthol of wild-type *Drosophila melanogaster* lines was differently affected depending on the exposure procedure. After many generations, flies forcibly kept on menthol-rich food consistently avoided oviposition on this substance, whereas ‘choice-line’ flies showed a decreased aversion to menthol, indifference or even attraction at some generations. This counterintuitive difference may explain – or reflect – some discrepancies noted between field and laboratory studies. If nature generally offers a broader choice of food resources than a controlled environment, wild animals need to constantly adapt their diet to environmental changes, and this is not, or less, the case for laboratory animals raised on a constant diet (Chu et al., 1995; Fritz and de Garine-Wichatitsky, 1996; Kraak, 1996; Sotka and Hay, 2002). It is possible that lines exposed to ever-changing food stimuli conserve a greater genetic variance than lines kept in a predictable food environment. For example, if two sources of food with low- and high-nutrient contents differently affect oviposition behaviour in two natural variant flies, the proximity of these two sources in nature may favour the co-existence of these two variants, together with the intraspecific genetic variance (McConnell, 2011). Conversely, the genetic variance related to sex-specific characters was not reduced in *D. melanogaster* natural lines after 50 laboratory generations (Houot et al., 2010). The generalist diet (and the short generation time) of this species may promote its rapid adaptation to food change, this explaining its cosmopolitan distribution. This contrasts with specialized-diet animals, which show no or little adaptation to novel food (linked with a reduced adaptive plasticity) such as in the case of strict plant-insect association (Agosta, 2006; Kühnle and Müller, 2011). Still in *Drosophila*, a shift in food preference was likely induced by a changed regulation in the gene coding for an odorant-binding protein (OBP57d/e) affecting the attraction and oviposition responses of *D. sechellia* flies to the odorant molecules emitted by its specific host fruit, *Morinda citrifolia* (Matsuo et al., 2007).

### Forced adaptation on menthol has no effect

Egg-laying site choice is a crucial decision-making process with regard to fitness (Yang et al., 2008). The ability of *D. melanogaster* females to evaluate the best available food source where to lay eggs depends both on its neural ability to determine food quality, but also on its experience (Sarin and Dukas, 2009; Anagnostou et al., 2010; Miller et al., 2011). In our hands, menthol induced a strong repulsive behaviour in M-forced females and this effect persisted after many – at least 52 – generations of forced exposure. The stability of this response indicates that the expression of genes underlying the perception of – and the oviposition response to – menthol is very robust linked maybe with the potential toxic effect induced by this substance.

Studies dealing with insects maintained during several generations on alternative sources of food reported contrasted behavioural effects. A chrysomelid beetle, *Phaedon cochleariae*, kept during 10 generations on a novel source of food showed no variation of food preference compared to the control line kept on the original food (Kühnle and Müller, 2011). Similarly, *D. melanogaster* lines kept during 35 generations on standard cornmeal–agar–yeast medium, banana, or tomato, did not change mate preference according to their nutritional status or that of their sex partner (Pavković-Lučić, 2009). Conversely, larvae of the cactophilic species *D. mojavensis* raised on laboratory food medium rapidly changed their adult behaviour (Brazner and Etges, 1993). The apparent discrepancy noted between these studies and others may be explained by the diversity of (*i*) the behaviours related to egg-laying (settling, oviposition, positional avoidance, mating, feeding) (Jaenike, 1986; Joseph et al., 2009), (*ii*) the exposure period (Barron and Corbet, 1999; Barron and Corbet, 2000), (*iii*) the diet (generalized in *D. melanogaster* (Ashburner, 1989) and specialized in *D. mojavensis* (Dethier, 1976)), or (*iv*) the experimental design (such as the distance between choice sites; Miller et al., 2011), the eggs of another female (Del Solar and Palomino, 1966); the degree of satiety (Dethier, 1976; Ryuda et al., 2008), and the choice between different sources of food (Sellier et al., 2011). Mice studies confirmed that the behavioural performance can be strongly impacted by the experimental design and by the laboratory environment (Crabbe et al., 1999). We are confident that our study, which was performed with genetically related flies raised and manipulated in similar conditions, allowed us to compare the impact of the ‘forced’ and ‘choice’ procedures on oviposition site-choice.

### The choice procedure affects oviposition in a non-linear manner

The choice procedure induced relatively fast changes: after 12 generations, the repulsive effect of menthol was significantly decreased in the M-choice line. If the decreased aversion of F12 flies of the M-choice line was somewhat expected, the strong reversal preference to menthol (e.g. the attraction shown at F20, F24, F25, F43, F45; supplementary material Fig. S1B) was unexpected. We do not know whether these sporadic changes are biologically meaningful. Possibly, this non-linear response between generations may reflect differential alteration of the genes underlying the multiple sensory systems integrating the perception of menthol (see below) and leading to the global behavioural response of the female. In any case, since this strongly contrasts with the highly stable (repulsive) response shown by forced lines, it suggests that the system underlying female oviposition choice was differently affected by our two experimental procedures.

If *Drosophila* oviposition behaviour is connected with other behaviours – such as attraction to food in *D. tripunctata* (Jaenike, 1986) and positional avoidance to acetic acid in *D. melanogaster* (Joseph et al., 2009) – then these behaviours depend on distinct genetic and chemosensory pathways. In particular, oviposition on acetic acid depends on the gustatory system whereas positional avoidance relies on olfaction (Joseph et al., 2009). Both olfactory and gustatory senses are successively required for optimal adult discrimination of sex pheromones (Everaerts et al., 2010). Menthol detection may rely at least on three sensory modalities: olfaction, taste and cold-sensing (Nagata et al., 2005). The fast and sporadic reversal of the response – from aversion to attraction – of the P-choice line suggests that these flies, which did not physically contact – but could nevertheless smell – menthol were somewhat exposed to this substance. Therefore, it is possible that our choice selection procedure has allowed us to successively select (1) ‘menthol-tasting’ genes (only in M-lines with physical contact to menthol), and (2) ‘menthol-smelling’ genes (necessary for olfactory site-choice in both in M- and P-lines). If this is true, the indifferent response to M-food would result of the counteracting effects resulting of the altered taste response combined with the unaltered olfactory response to menthol. On the other hand, attraction to M-food may result of the additive effects resulting of simultaneous alteration of taste, cold-sensing and olfactory responses to menthol. Otherwise, we found that the two choice lines showing the most altered response to menthol (F24+F25) had an unaltered response to two other substances with either an aversive or an appetitive effect. This indicates that our choice procedure induced relatively specific effects on ‘menthol-responding’ genes, but we do not know the sensory modalities involved. Furthermore, our study cannot provide any support to the controversial experiments involving preimaginal conditioning with menthol in flies (Thorpe, 1939; Alloway, 1972; Barron and Corbet, 1999), since our experiments involved different developmental exposures to menthol (see also the discussion above).

### The choice procedure also affects fecundity

The variation of fecundity observed during the course of the choice experiment may be a side effect of our choice procedure. The positive relationship between the variation of both the MPI and EPF in F12 females of different ages (Fig. 4) suggests that the increased fecundity of M-choice females principally results of the increased fecundity on M-food (whereas the fecundity on plain food remains unchanged). Such increased fecundity on menthol may be related to the earlier peak of fecundity shown by the two choice lines, and particularly the M-choice line (Fig. 4B). Since fecundity rapidly increased (after only one generation; Table 1; supplementary material Fig. S3), this phenomenon may be a result of a biased sampling of the early progeny laid by the most precocious egg-laying females. *D. pseudoobscura* lines selected for reduced dispersion of oviposition sites also showed a rapid fecundity increase (2–3 generations) in ‘high dispersion’ lines (Del Solar, 1968). Conversely, the relatively stable fecundity of ‘forced’ females (at least during the first 9 generations) can be explained by the absence of bias in egg collection used to induce the next generation.

In summary, our study reveals a striking difference in oviposition preference on menthol-rich food according to the experimental procedure: the choice procedure induced a faster – but non-permanent – change whereas the forced procedure had no major effect. Our next goal will consist of characterizing some of the genetic factors and the neural pathways underlying the change of the behavioural response to menthol in choice lines.

## Supplementary Material

Supplementary Material

## References

[b1] Agosta S. J. (2006). On ecological fitting, plant-insect associations, herbivore host shifts, and host plant selection. Oikos 114, 556–565 10.1111/j.2006.0030-1299.15025.x

[b2] Agresti A. (2002). Categorical Data Analysis, 2nd edition 717 New York, NY: Wiley.

[b3] Allemand R. Boulétreau-Merle J. (1989). Correlated responses in lines of Drosophila melanogaster selected for different oviposition behaviors. Experientia 45, 1147–1150 10.1007/BF01950184 2513224

[b4] Alloway T. M. (1972). Learning and memory in insects. Annu. Rev. Entomol. 17, 43–56 10.1146/annurev.en.17.010172.000355

[b5] Anagnostou C. LeGrand E. A. Rohlfs M. (2010). Friendly food for fitter flies? – Influence of dietary microbial species on food choice and parasitoid resistance in Drosophila. Oikos 119, 533–541 10.1111/j.1600-0706.2009.18001.x

[b6] Ashburner M. (1989). Drosophila: A Laboratory Handbook 1375 Cold Spring Harbor, NY: Cold Spring Harbor Laboratory Press.

[b7] Barron A. B. Corbet S. A. (1999). Preimaginal conditioning in Drosophila revisited. Anim. Behav. 58, 621–628 10.1006/anbe.1999.1169 10479377

[b8] Barron A. B. Corbet S. A. (2000). Behavioural induction in Drosophila: timing and specificity. Entomol. Exp. Appl. 94, 159–171 10.1046/j.1570-7458.2000.00616.x

[b9] Bertin A. Calandreau L. Arnould C. Lévy F. (2012). The developmental stage of chicken embryos modulates the impact of in ovo olfactory stimulation on food preferences. Chem. Senses 37, 253–261 10.1093/chemse/bjr101 22080043

[b10] Brazner J. C. Etges W. J. (1993). Pre-mating isolation is determined by larval rearing substrates in cactophilic Drosophila mojavensis. II. Effects of larval substrates on time to copulation, mate choice and mating propensity. Evol. Ecol. 7, 605–624 10.1007/BF01237824

[b11] Brown L. van der Ouderaa F. (2007). Nutritional genomics: food industry applications from farm to fork. Br. J. Nutr. 97, 1027–1035 10.1017/S0007114507691983 17506913

[b12] Chu C. C. Henneberry T. J. Cohen A. C. (1995). Bemesia argentifolii (Homoptera: Aleyrodidae): host preference and factors affecting oviposition and feeding site preference. Environ. Entomol. 24, 354–360.

[b13] Crabbe J. C. Wahlsten D. Dudek B. C. (1999). Genetics of mouse behavior: interactions with laboratory environment. Science 284, 1670–1672 10.1126/science.284.5420.1670 10356397

[b14] Del Solar E. (1968). Selection for and against gregariousness in the choice of oviposition sites by Drosophila pseudoobscura. Genetics 58, 275–282. 5664388 10.1093/genetics/58.2.275 PMC1211859

[b15] Del Solar E. Palomino H. (1966). Choice of oviposition in Drosophila melanogaster. Am. Nat. 100, 127–133 10.1086/282406

[b16] Dethier V. G. (1976). The Hungry Fly. A Physiological Study of the Behavior Associated with Feeding 489 Oxford: Harvard University Press.

[b17] Dierick H. A. Greenspan R. J. (2006). Molecular analysis of flies selected for aggressive behavior. Nat. Genet. 38, 1023–1031 10.1038/ng1864 16906161

[b18] Dukas R. (2008). Evolutionary biology of insect learning. Annu. Rev. Entomol. 53, 145–160 10.1146/annurev.ento.53.103106.093343 17803459

[b19] Everaerts C. Lacaille F. Ferveur J. F. (2010). Is mate choice in Drosophila males guided by olfactory or gustatory pheromones? Anim. Behav. 79, 1135–1146 10.1016/j.anbehav.2010.02.013

[b20] Fougeron A. S. Farine J. P. Flaven-Pouchon J. Everaerts C. Ferveur J. F. (2011). Fatty-acid preference changes during development in Drosophila melanogaster. PLoS ONE 6, e26899 10.1371/journal.pone.0026899 22046401 PMC3203165

[b21] Fritz H. de Garine-Wichatitsky M. (1996). Foraging in a social antelope: effects of group size on foraging choices and resource perception in impala. J. Anim. Ecol. 65, 736–742 10.2307/5672

[b22] Gerber B. Stocker R. F. Tanimura T. Thum A. S. (2009). Smelling, tasting, learning: *Drosophila* as a study case. Chemosensory Systems in Mammals, Fishes and Insects (Results and Problems in Cell Differentiation) Korsching S Meyerhof W, ed1–47 Berlin; Heidelberg: Springer-Verlag. 10.1007/400_2008_9 19145411

[b23] Greenspan R. J. (2001). The flexible genome. Nat. Rev. Genet. 2, 383–387 10.1038/35072018 11331904

[b24] Houot B. Svetec N. Godoy-Herrera R. Ferveur J. F. (2010). Effect of laboratory acclimation on the variation of reproduction-related characters in Drosophila melanogaster. J. Exp. Biol. 213, 2322–2331 10.1242/jeb.041566 20543131

[b25] Houot B. Fraichard S. Greenspan R. J. Ferveur J. F. (2012). Genes involved in sex pheromone discrimination in Drosophila melanogaster and their background-dependent effect. PLoS ONE 7, e30799 10.1371/journal.pone.0030799 22292044 PMC3264623

[b26] Jaenike J. (1986). Genetic complexity of host-selection behavior in Drosophila. Proc. Natl. Acad. Sci. USA 83, 2148–2151 10.1073/pnas.83.7.2148 16593679 PMC323248

[b27] Joseph R. M. Devineni A. V. King I. F. Heberlein U. (2009). Oviposition preference for and positional avoidance of acetic acid provide a model for competing behavioral drives in Drosophila. Proc. Natl. Acad. Sci. USA 106, 11352–11357 10.1073/pnas.0901419106 19541615 PMC2698888

[b28] Kent L. M. Worsley A. (2009). Trends in BMI, diet and lifestyle between 1976 and 2005 in North Sydney. Asia Pac. J. Clin. Nutr. 18, 453–461. 19786395

[b29] Kraak S. B. M. (1996). Female preference and filial cannibalism in Aidublennius sphynx (Teleostei, Blenniidae); a combined field and laboratory study. Behav. Processes 36, 85–97 10.1016/0376-6357(95)00019-4 24896420

[b30] Kühnle A. Müller C. (2011). Responses of an oligophagous beetle species to rearing for several generations on alternative host-plant species. Ecol. Entomol. 36, 125–134 10.1111/j.1365-2311.2010.01256.x

[b31] Matsuo T. Sugaya S. Yasukawa J. Aigaki T. Fuyama Y. (2007). Odorant-binding proteins OBP57d and OBP57e affect taste perception and host-plant preference in Drosophila sechellia. PLoS Biol. 5, e118 10.1371/journal.pbio.0050118 17456006 PMC1854911

[b32] McConnell M. W. (2011). Exploring the Role of the foraging Gene on Egg-Laying Preferences in Drosophila melanogaster 68 MSc thesis, University of Toronto, Old Toronto, Toronto, ON, Canada

[b33] Melcher C. Bader R. Pankratz M. J. (2007). Amino acids, taste circuits, and feeding behavior in Drosophila: towards understanding the psychology of feeding in flies and man. J. Endocrinol. 192, 467–472 10.1677/JOE-06-0066 17332516

[b34] Mennella J. A. Jagnow C. P. Beauchamp G. K. (2001). Prenatal and postnatal flavor learning by human infants. Pediatrics 107, E88 10.1542/peds.107.6.e88 11389286 PMC1351272

[b35] Miller P. M. Saltz J. B. Cochrane V. A. Marcinkowski C. M. Mobin R. Turner T. L. (2011). Natural variation in decision-making behavior in Drosophila melanogaster. PLoS ONE 6, e16436 10.1371/journal.pone.0016436 21283727 PMC3024433

[b36] Nagata H. Dalton P. Doolittle N. Breslin P. A. (2005). Psychophysical isolation of the modality responsible for detecting multimodal stimuli: a chemosensory example. J. Exp. Psychol. Hum. Percept. Perform. 31, 101–109 10.1037/0096-1523.31.1.101 15709866

[b37] Ogueta M. Cibik O. Eltrop R. Schneider A. Scholz H. (2010). The influence of Adh function on ethanol preference and tolerance in adult Drosophila melanogaster. Chem. Senses 35, 813–822 10.1093/chemse/bjq084 20739429

[b38] Papaj D. R. Prokopy R. J. (1989). Ecological and evolutionary aspects of learning in phytophagous insects. Annu. Rev. Entomol. 34, 315–350 10.1146/annurev.en.34.010189.001531

[b39] Pavković-Lučić S. (2009). Is there ethological isolation among Drosophila melanogaster strains reared for more than 35 generations on different food? Arch. Biol. Sci. 61, 105–112 10.2298/ABS0901105P

[b40] Ryuda M. Shimada K. Koyanagi R. Azumi K. Tanimura T. Hayakawa Y. (2008). Analysis of hunger-driven gene expression in the Drosophila melanogaster larval central nervous system. Zoolog. Sci. 25, 746–752 10.2108/zsj.25.746 18828662

[b41] Sarin S. Dukas R. (2009). Social learning about egg-laying substrates in fruitflies. Proc. Biol. Sci. 276, 4323–4328 10.1098/rspb.2009.1294 19759037 PMC2817106

[b42] Sellier M. J. Reeb P. Marion-Poll F. (2011). Consumption of bitter alkaloids in Drosophila melanogaster in multiple-choice test conditions. Chem. Senses 36, 323–334 10.1093/chemse/bjq133 21173029

[b43] Sotka E. E. Hay M. E. (2002). Geographic variation among herbivore populations in tolerance for a chemically rich seaweed. Ecology 83, 2721–2735 10.1890/0012-9658(2002)083[2721:GVAHPI]2.0.CO;2

[b44] Stewart J. E. Feinle-Bisset C. Keast R. S. J. (2011). Fatty acid detection during food consumption and digestion: Associations with ingestive behavior and obesity. Prog. Lipid Res. 50, 225–233 10.1016/j.plipres.2011.02.002 21356242

[b45] Takamura T. Fuyama Y. (1980). Behavior genetics of choice of oviposition sites in Drosophila melanogaster. I. Genetic variability and analysis of behavior. Behav. Genet. 10, 105–120 10.1007/BF01067322 6775626

[b46] Thorpe W. H. (1939). Further studies on pre-imaginal olfactory conditioning in insects. Proc. R. Soc. B 127, 424–433 10.1098/rspb.1939.0032

[b47] Toma D. P. White K. P. Hirsch J. Greenspan R. J. (2002). Identification of genes involved in Drosophila melanogaster geotaxis, a complex behavioral trait. Nat. Genet. 31, 349–353 10.1038/ng893 12042820

[b48] Yang C. H. Belawat P. Hafen E. Jan L. Y. Jan Y. N. (2008). Drosophila egg-laying site selection as a system to study simple decision-making processes. Science 319, 1679–1683 10.1126/science.1151842 18356529 PMC2581776

[b49] Yeomans M. R. Leitch M. Gould N. J. Mobini S. (2008). Differential hedonic, sensory and behavioral changes associated with flavor-nutrient and flavor-flavor learning. Physiol. Behav. 93, 798–806 10.1016/j.physbeh.2007.11.041 18201736

[b50] Zhang Y. Lu H. Bargmann C. I. (2005). Pathogenic bacteria induce aversive olfactory learning in Caenorhabditis elegans. Nature 438, 179–184 10.1038/nature04216 16281027

