# Supplementary material for: Choice alters Drosophila oviposition site preference on menthol
Source: Biol Open. 2013 Sep 29;3(1):22–8. doi: 10.1242/bio.20136973 (PMC3892157; doi:10.1242/bio.20136973)
Supplement: Supplementary Material [file supp_3_1_22__index.html]

Choice alters Drosophila oviposition site preference on menthol — Choice alters Drosophila oviposition site preference on menthol — Supplementary Material 

# Choice alters *Drosophila* oviposition site preference on menthol

## bio.20136973 Supplementary Material

**Files in this Data Supplement:**

- Supplementary Material - Dehbia Abed-Vieillard et al. doi: 10.1242/bio.20136973
