# Supplementary material for: Choice alters Drosophila oviposition site preference on menthol
Source: Biol Open. 2013 Sep 29;3(1):22–8. doi: 10.1242/bio.20136973 (PMC3892157; doi:10.1242/bio.20136973)
Supplement: Supplementary Material [file supp_bio.20136973_bio.20136973-s1.pdf]

Biology Open

Supplementary Material

Dehbia Abed-Vieillard et al. doi: 10.1242/bio.20136973

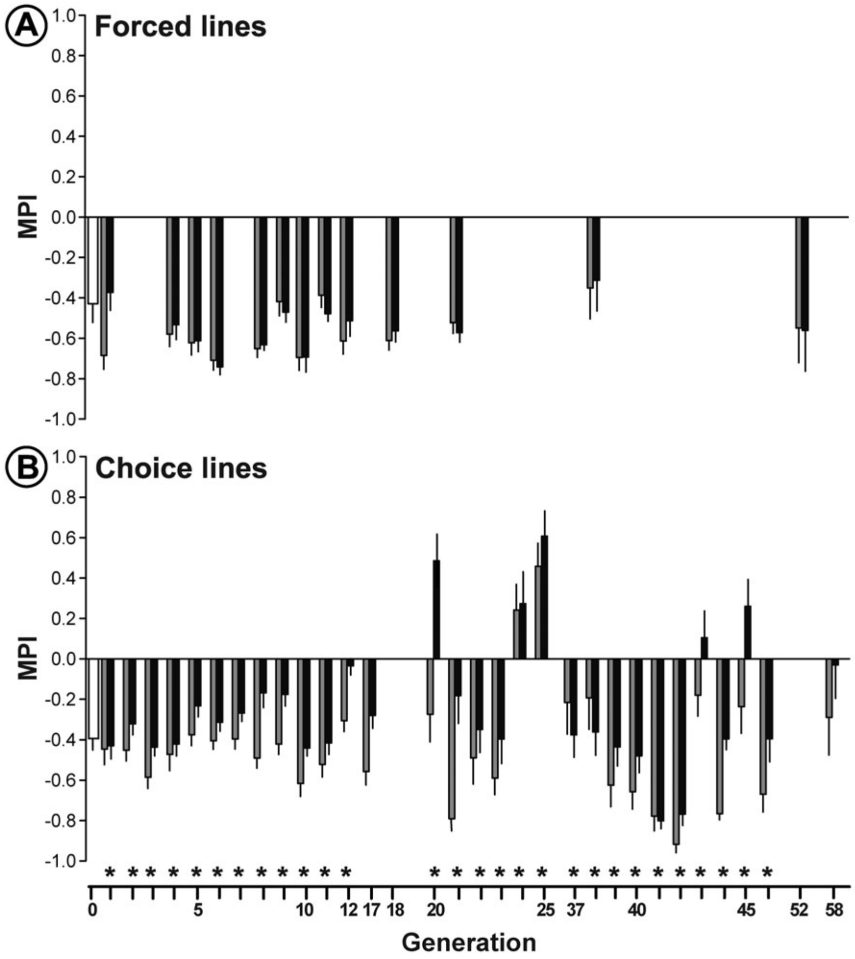

Fig. S1. Oviposition site preference on menthol in forced lines (A) and choice lines (B) during more than 50 generations. Bars represent the mean (± SEM) for the oviposition site preference (MPI) in lines either maintained on P-food (P-line; shaded bars), or on M-food (M-line, filled bars). The performance of both forced lines was measured at several generations between F1 and F58 together with that of the control (unselected) line at the beginning (F0; empty bar). The stars indicate the generations undergoing the choice procedure for the choice lines.

Biology Open

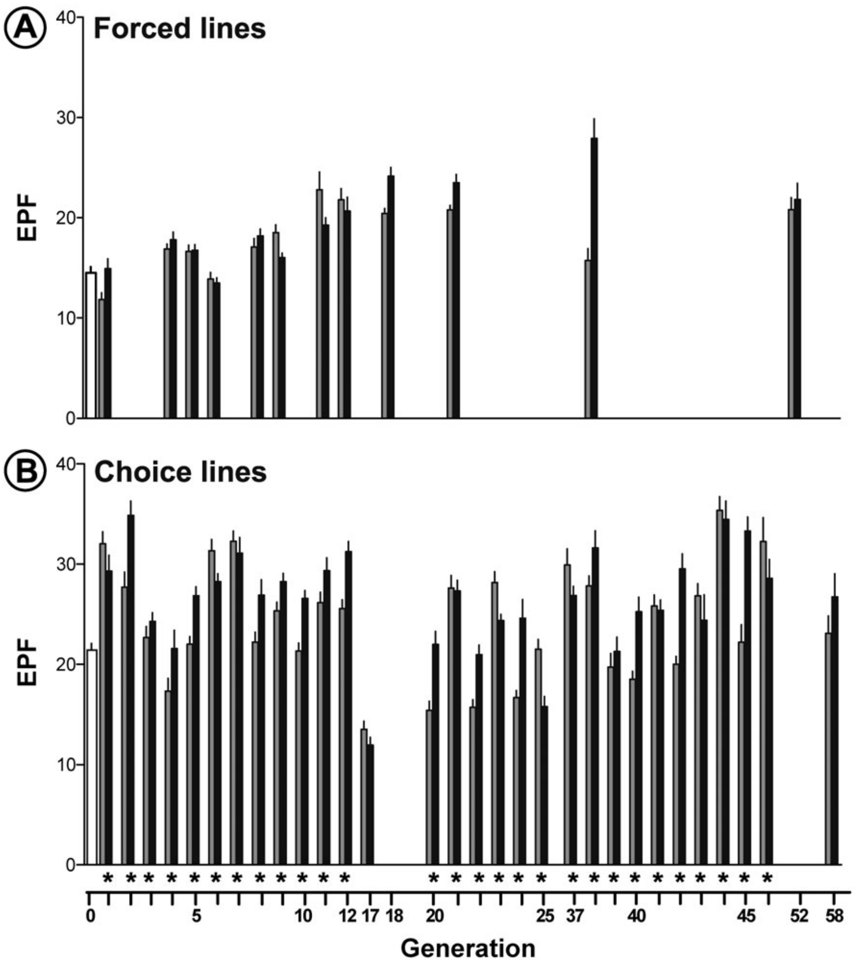

Fig. S2. Eggs laid per female (EPF) on menthol in forced lines (A) and choice lines (B) during more than 50 generations. Bars represent the mean (± SEM) for the EPF in lines either maintained on P-food (P-line; shaded bars), or on M-food (M-line, filled bars). The performance of both forced lines was measured at several generations between F1 and F58 together with that of the control (unselected) line at the beginning (F0; empty bar). The stars indicate the generations undergoing the choice procedure for the choice lines.

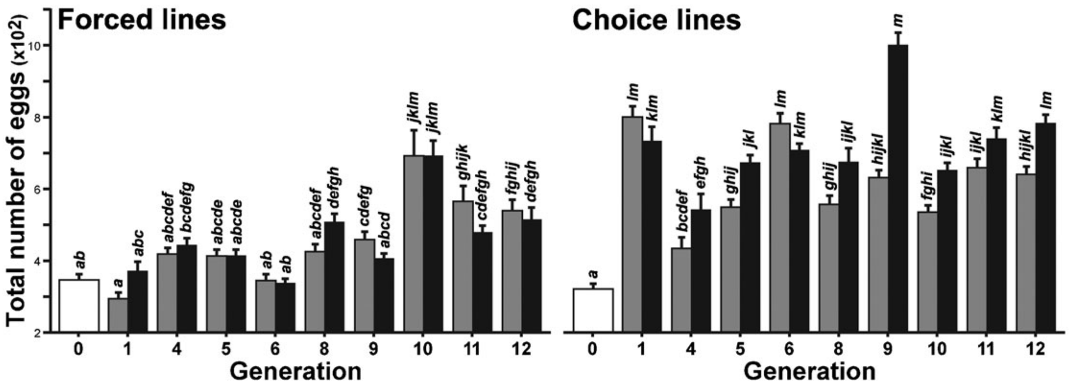

Fig. S3. Total number of eggs laid by choice-line females between F1 and F12 generations. Bars represent the mean (± SEM) for the total number of eggs laid by 25 females from lines either maintained (forced or choice procedure) on P-food (P-line; shaded bars) or on M-food (M-line, filled bars). Letters indicate the significant differences (p=0.05) with a Kruskal–Wallis test (K_{37df}=554.46, p<0.0001; n=5–34).

Table S1. Statistical analysis of 'choice' and 'forced' lines for menthol preference index (MPI) and eggs per females (EPF) during more than 50 generations. Data were analyzed with an ANCOVA with 'MPI' and 'EPF' as dependent variables, 'Generation' and 'Type of food' as quantitative and qualitative independent variables, respectively. The Ryan–Einot–Gabriel–Welsch (REGWQ) test was used for multiple paired comparison. The signs (<, =, and >) show the hierarchy of values for the three lines (C, P and M are the control, P- and M-lines, respectively).

Line	Generations	MPI	EPF
Forced line	–	C=P=M<br>$F_{3,443}$=2.238 p=0.083	C<P<M<br>$F_{3,443}$=21.562 p<0.0001
	F0–F12	C=P; C=M; P<M<br>$F_{3,637}$=14.070 p<0.0001	C<P<M<br>$F_{3,637}$=53.573 p<0.0001
Choice line	[F0–F12]+[F20–F25]	P<C=M<br>$F_{3,948}$=27.825 p<0.0001	C<P<M<br>$F_{3,948}$=75.841 p<0.0001
	[F0–F12]+[F37–F46]	C<P<M<br>$F_{3,1149}$=19.15 p<0.0001	C<P<M<br>$F_{3,1149}$=15.478 p<0.0001

Biology Open
